# Emerging Roles of Rivastigmine Derivatives Bearing Antioxidant Motifs as Multi-Target Agents for the Management of Neurodegenerative Diseases

**DOI:** 10.3390/ijms27083637

**Published:** 2026-04-19

**Authors:** Inês Dias, Catarina Guerreiro-Oliveira, Inês Melo-Marques, Sandra M. Cardoso, Rita C. Guedes, Ismael Carvalho, Teresa Rocha, Daniel Chavarria, Sílvia Chaves, M. Amélia Santos

**Affiliations:** 1 Centro de Química Estrutural, Institute of Molecular Sciences, Departamento de Engenharia Química, Instituto Superior Técnico, Universidade de Lisboa, Av. Rovisco Pais 1, 1049-001 Lisboa, Portugal; 2 CNC-UC, Center for Neuroscience and Cell Biology, Universidade de Coimbra, 3004-504 Coimbra, Portugal; 3 Centre for Innovative Biomedicine and Biotechnology, University de Coimbra, 3004-504 Coimbra, Portugal; 4 FMUC, Faculdade de Medicina, Universidade de Coimbra, 3004-504 Coimbra, Portugal; 5 Research Institute for Medicines (iMed.ULisboa), Faculdade de Farmácia, Universidade de Lisboa, Av. Prof. Gama Pinto, 1649-003 Lisboa, Portugal; 6 RISE-Health, Department of Biomedicine, Pharmacology and Therapeutics Unit, Faculty of Medicine, University of Porto, Alameda Prof. Hernâni Monteiro, 4200-319 Porto, Portugal

**Keywords:** neurodegenerative diseases, rivastigmine hybrids, antioxidants, biometal chelation, amyloid-β aggregation, monoamine oxidase

## Abstract

Neurodegenerative disorders (NDs), such as Alzheimer’s and Parkinson’s diseases (AD and PD), despite having different main neuropathological hallmarks, share several interconnected aetiologic mechanisms and lack effective disease-modifying treatments. The multifactorial nature of these diseases has encouraged the development of new drugs such as multi-target-directed ligands (MTDLs). In this work, an anti-AD drug (rivastigmine, RIV) was fused and conjugated with a series of antioxidant scaffolds to obtain a small library of RIV–antiox hybrids. In addition to inhibitory activity towards both cholinesterases, these hybrids exhibited radical scavenging activity, inhibition of Aβ aggregation, and neuroprotection against cell death induced in AD models. The relevant anti-AD properties already found for these hybrids challenged us to also assess their capacity to modulate and interfere with ROS-associated harmful dysfunctions, namely in the dysregulation of biometal ions (Fe^3+^, Cu^2+^, and Zn^2+^) and upregulation of monoamine oxidases (MAOs). In particular, the capacity of the hybrids for metal chelation and inhibition of Cu-induced Aβ aggregation and MAO isoforms was evaluated, as well as their neuroprotection capacity in cell models of PD. Overall, some of these RIV hybrids appear as lead compounds for the development of novel multifunctional agents against NDs.

## 1. Introduction

Neurological disorders are now the leading cause of disability in the world. Among them, Alzheimer’s disease (AD) is the most prevalent of these diseases, progressively affecting memory and cognitive functions, i.e., dementia, while Parkinson’s disease (PD) is the second most common neurodegenerative disease (ND), featuring mainly progressive impairment of motor functions but also a mild cognitive deficit [1,2]. AD and PD are age-related NDs; with life expectancy increasing, the number of patients will continue to increase, placing massive burdens on healthcare systems and society. Despite significant scientific investment worldwide, the available treatments fail to delay or halt the progression of NDs, a feature mainly attributed to the complex and intricate multifactorial nature of these diseases.

The main neuropathological hallmarks of AD and PD are based in abnormal protein folding and aggregation, leading to toxic insoluble protein and peptide aggregates in patients’ brains, namely due to the accumulation of amyloid-β (Aβ) in extracellular senile plaques and of tau protein in intracellular neurofibrillary tangles (NFTs), which is associated with AD [3], and accumulation of α-synuclein (α-syn) in Lewy bodies (LBs), which is associated with PD [4]. Besides these differences in the main pathophysiological hallmarks, the brains of both AD and PD patients present dramatic loss of specific neuronal populations, namely cholinergic and dopaminergic neurons in AD and PD, respectively. Another significant risk factor that has been linked to the onset and progression of these NDs is increased oxidative stress (OS), namely due to the overproduction of reactive oxygen species (ROS) [5] and to ROS-associated harmful dysfunctions, such as the dysregulation of redox-active biometal ions (Cu and Fe), which can generate ROS (Fenton Reaction) [6]; however, some biometals can also promote neuropeptide and protein aggregation, namely Cu (interlinked with Zn) in Aβ of AD [7] and Fe in α-syn of PD Lewy bodies [6]. Moreover, oxidant activity acts as a bridge between nervous and immune systems, since oxidative–inflammatory situations caused by anxiety or the production of ROS by phagocytic immune cells to destroy pathogens lead to neuronal death and degeneration as well as to faster immunosenescence. In addition, there is an upregulation of monoamine oxidases (MAOs), which oxidatively deaminate monoamines such as dopamine and serotonin, with concomitant generation of neurotoxic hydrogen peroxide (H_2_O_2_) [8].

A great variety of novel drug candidates for these neurological disorders have been explored worldwide over the years but without effective disease-modifying effects [9,10]. The conventional clinical drugs have largely underperformed, without addressing the cause and cure of these diseases. They have been mainly focused on the alleviation of symptoms, by compensating for the loss of neurotransmitters, namely acetylcholine in AD, by approved cholinesterase inhibitors (ChEis), such as donepezil, rivastigmine and galantamine [11], and dopamine in PD, by dopamine precursors (e.g., L-dopa) or agonists [12], though rivastigmine is also used to treat cognitive slowing in PD [13]. Anti-amyloid drugs for AD, based on monoclonal antibodies (e.g., aducanumab and lecanemab), have also been the focus of recent investigations, but, due to some lack of efficacy and safety concerns, only slight advances have been reported in Aβ therapy [14].

Since therapeutic strategies based on targeting a single causative agent or risk factor are insufficient to treat AD and PD, there is an escalating challenge to find effective drugs for clinics. The vast evidence supporting ND multiple pathogenesis warrants a paradigm shift from traditional single-target drugs to multi-target-directed ligands (MTDLs), relying on their capacity to simultaneously modulate multiple relevant neurobiological targets and improve therapeutic efficacy. Therefore, over the last two decades, the development of MTDL-based drugs for neurodegenerative diseases, namely AD, has been the focus of quite intensive investigation worldwide [15,16,17,18].

The design of MTDLs has been mainly based on combining two or more key pharmacologically active scaffolds into a single molecular entity with the aim of obtaining additive or even synergistic effects. Among different approaches towards new MTDLs, the derivatization of pharmacophoric moieties of already approved (“old”) drugs has been the object of major interest, with the hope that the new hybrid drugs could keep former biological activities and also mitigate attritions in translational processes. Following this rationale, different classes of hybrids have been explored by combining the active scaffold of approved anti-AD ChE inhibitor (AChEi) drugs (e.g., tacrine, donepezil, and rivastigmine) with other bioactive moieties to enable additional therapeutic benefits, such as the inhibition of Aβ aggregation, antioxidant activity, the modulation of biometals, and the inhibition of MAOs [19,20,21,22,23,24,25].

Among the ChEi anti-AD drugs, scaffolds based on tacrine have been by far the most explored, followed by those from donepezil, while rivastigmine has been the least explored, possibly because it is the most recent and least potent ChE inhibitor. However, the fact that rivastigmine possesses dual cholinesterase (AChE and BChE) inhibitory activity brings remarkable advantages to the corresponding hybrids, and so this dual inhibitory capacity has been recognized as an important key therapeutic target of AD (and other dementias such as PD) [26]. Therefore, several rivastigmine-based MTDLs have recently been developed, exhibiting multifunctional properties, including antioxidant activity, inhibition of ChEs and MAOs, and Aβ aggregation as well as metal-chelating capacity [25,27,28,29]. The best-known example of this type of compound is Ladostigil, a dual inhibitor of CHEs and MAOs that has entered into clinical trials [30].

**Scheme 1:**
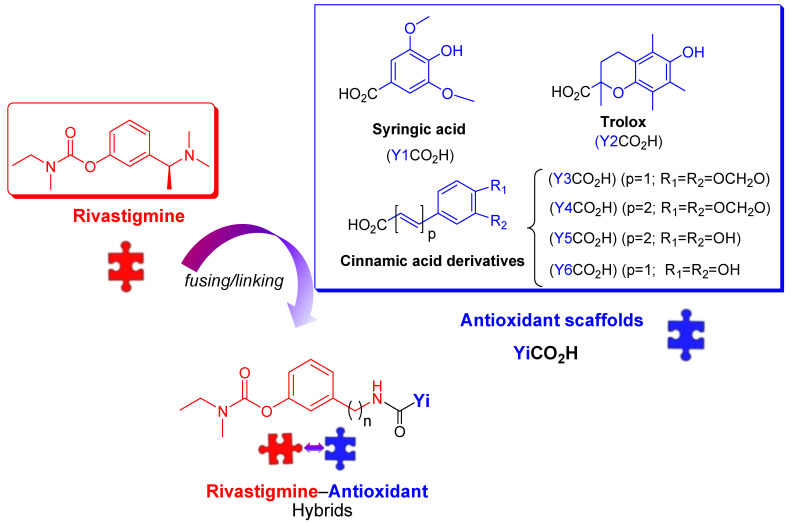
Design strategy for the hybrids from rivastigmine templates and antioxidant scaffolds. General structures of the rivastigmine–antioxidant (RIV–antiox) hybrids.

Taking into account that oxidative stress and neuroinflammation play pivotal roles in the etiology and progression of neurodegenerative diseases [31,32], we recently developed and studied a series of MTDLs incorporating rivastigmine fragments (RIV) hybridized with a set of different scaffolds with important antioxidant (or pro-antioxidant) properties (namely Trolox, syringic acid, and dihydroxyl derivatives of cinnamic acid and cinnamylidene acetic acid (see Scheme 1)) [33]. The relevant multipotent anti-AD properties revealed by these hybrids, e.g., anti-oxidation and inhibition of ChEs and Aβ aggregation, as well as regeneration of neuronal cells stressed by AD models such as Aβ and iron/ascorbate oxidative, challenged us to conduct complementary studies to evaluate additional neuroprotective properties of these hybrids.

In this study, we focused on the evaluation of the capacity of those hybrids to modulate and interfere with ROS-associated harmful dysfunctions, namely due to the dysregulation of biometal ions or upregulation of monoamine oxidases (MAOs). In fact, as stated above, Cu and Zn are known to promote protein misfolding such as Aβ aggregation of AD, while Cu and Fe also promote ROS generation. Thus, we evaluated the effect of the hybrids on the inhibition of Cu-induced Aβ aggregation. To gain a further insight into the capacity of these metal-chelating hybrids to interfere with metal-related disfunctions, we performed a quantitative evaluation of the metal-chelating capacity of one active inhibitor of Aβ aggregation (**4BY1**) with Cu(II), Zn(II), and Fe(III). This compound was selected because qualitative metal-chelating studies with other analogs of the most active hybrids (**4AY5** and **4AY6**), namely containing ChEis coupled to identical cinnamate coordinating cores, were previously reported [34,35,36]. We also assessed the capacity of our compounds to inhibit both monoamine oxidase (MAO) isoforms (MAO-A and MAO-B) and complemented the experimental results with docking simulations aiming to shed some light on the ligand–enzyme interactions and structure–activity relationships. Finally, the neuroprotective effects of the rivastigmine-derived compounds against 1-methyl-4-phenylpyridinium (MPP^+^)-induced damage in human neuroblastoma (SH-SY5Y) cells were assessed, with an in vitro cell model of dopaminergic neuron death in PD.

## 2. Results and Discussion

### 2.1. Biometal Chelation Studies

It is well known that the dyshomeostasis of important endogenous biometals (Fe, Cu, and Zn) in the brain is connected to AD onset, due to several processes such as protein/peptide modification, Aβ aggregation, and ROS generation with subsequent neurotoxic and inflammatory responses [37,38]. Under the strategy of developing potential multi-target anti-neurodegenerative drugs able to modulate biometals, some of the herein studied rivastigmine-derived hybrids include moieties with metal-chelating ability, namely those derived from syringic acid (**4AY1**, **4BY1**, and **4CY1**) or cinnamic acid (**4AY5**) and cinnamylidene acetic acid (**4AY6**) with dihydroxyl groups.

Catechol and catechol-derived ligands have been largely studied in terms of their metal chelation ability [39,40,41], and although the hybrids bearing cinnamate derivatives have been qualitatively evaluated [34,35,36], the metal complexation studies regarding syringic acid are scarce [42,43,44,45]. Therefore, we decided to study the metal chelation of a model compound among the hybrids bearing syringic moieties. In particular, **4BY1** was chosen to be evaluated as a metal chelator of Fe^3+^, Cu^2+^, and Zn^2+^, due to its most promising Aβ inhibitory potential among the syringic acid-derived hybrids (**4AY1**, **4BY1**, and **4CY1**). Therefore, the acid-base properties and metal (Fe, Cu, and Zn)-chelating capacity of **4BY1** were studied by using pH potentiometric and UV–vis spectrophotometric titrations in a 10% DMSO/water medium due to some water-solubility constraints. The subsequent equilibrium models were obtained by fitting analysis of the experimental data, as detailed in Section 3.

#### 2.1.1. Acid-Base Properties

**Figure 1:**
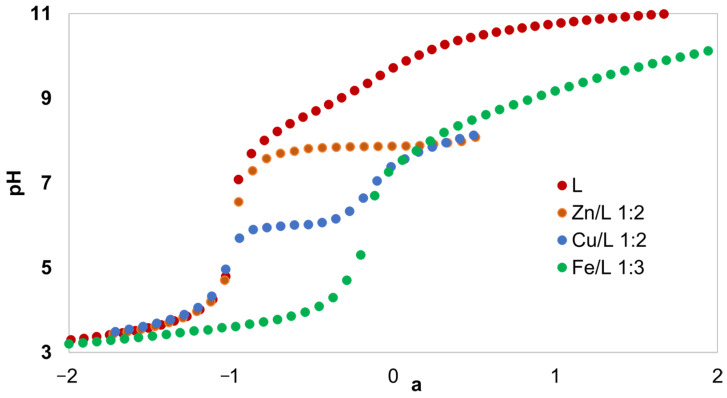
Potentiometric titration curves of **4BY1** and respective M/L systems (*C*_L_ = 6.0 × 10^−4^ M); *a* represents moles of added base per mole of ligand.

Firstly, the protonation constant of the phenolate group was determined by both potentiometric (see Figure 1) and spectrophotometric titrations, and the value obtained was subsequently included in the metal complexation models. The compound was isolated in its deprotonated form (L^−^, *a* = 0 in Figure 1), becoming neutral (HL) with the protonation of the *O*-phenol atom.

The obtained protonation constants are shown in Table 1, the calculated values—8.61 and 8.627—being quite similar for both the experimental techniques used. Comparing these values with those obtained in the literature for the corresponding phenolic group of syringic acid (log *K* = 8.73), vanillic acid (log *K* = 8.70) or ferulic acid (log *K* = 8.7) [46], the slight differences can be mainly explained by the different para substituents of the phenol, but also by the experimental conditions used (medium, temperature, and ionic strength).

#### 2.1.2. Biometal Complexation Assays

**Figure 2:**
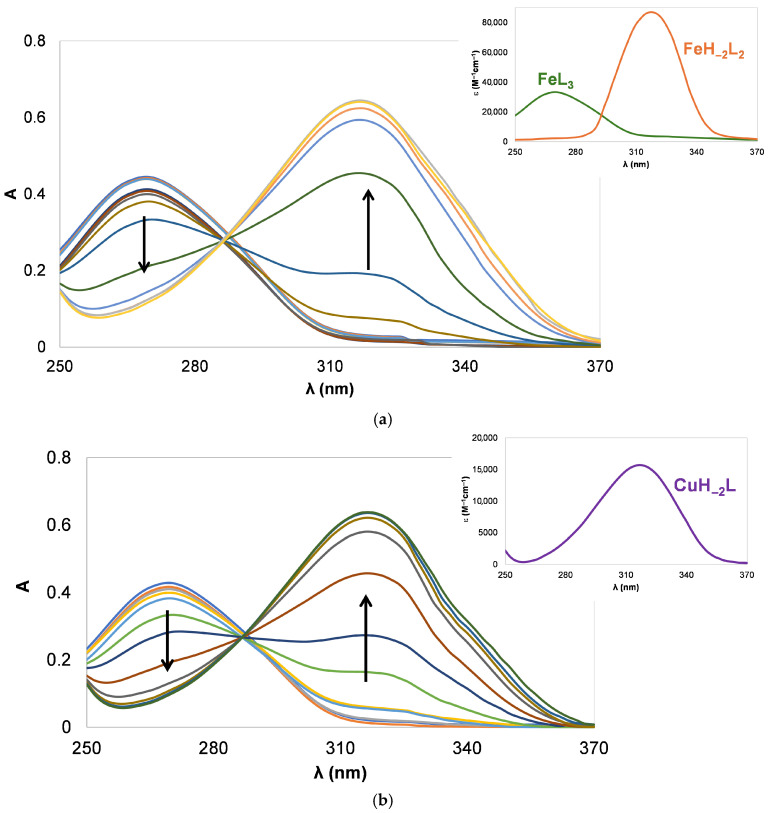
Spectrophotometric absorption spectra of the systems (**a**) Fe^3+^/**4BY1** 1:3 (3.23 < pH < 10.85) and (**b**) Cu^2+^/**4BY1** 1:2 (4.94 < pH < 11.02) (*C*_L_ = 4 × 10^−5^ M). Outsets contain calculated spectra by Psequad and the arrows show the progression of the spectra with pH increase.

The biometal complexation models were obtained from the treatment of the resultant experimental data from pH potentiometric titrations in the case of the M/**4BY1** systems (M = Fe, Cu, and Zn; Figure 1) and from spectrophotometric titrations for the Fe^3+^ and Cu^2+^ systems (Figure 2), under different metal-to-ligand ratios (M/L 1:1 and 1:2, M = Cu and Zn; M/L 1:1, 1:2, and 1:3, M = Fe). Figure 1 evidences a change in the deprotonation profile of the ligand, for −1 < *a* < 0 and for all the system M/**4BY1** (M = Fe, Cu and Zn), as well as for a < −1 in the case of the 1:3 Fe^3+^/**4BY1** system. This observation is in accordance with the involvement of the *O*-phenolic atom in the coordination of the metal ions, for a pH above ca. 5–6 in the case of the Cu^2+^ and Zn^2+^ systems and for quite an acidic pH in the case of the Fe^3+^ complexes, with the following relative stability order: Fe > Cu > Zn.

Figure 2 includes the absorbance spectra of the 1:3 Fe^3+^/**4BY1** and 1:2 Cu^2+^/**4BY1** systems and also the respective calculated individual spectra. The collected spectrophotometric data are in the same wavelength range as that of **4BY1**, since ligand-to-metal charge-transfer (CT) bands for the Fe^3+^ and Cu^2+^ systems should appear in the visible wavelength range, but, under the experimental diluted conditions used, their low intensity excluded their use in the equilibrium studies.

Table 1 presents the obtained values for the metal stability constants of **4BY1**, showing quite simple complexation models for all the systems, including only one 1:1 species (ligand–metal–hydroxo, MH_−2_L = M(OH)_2_L) for the complexation with Cu^2+^ and Zn^2+^, while two complexes (FeH_−2_L_2_ and FeL_3_) are found in the case of the iron complexation model. In Figure 2a it is possible to see that the wavelength corresponding to the maximum absorbance of the calculated spectra of FeL_3_ (270 nm) has a lower value than that of the species FeH_−2_L_2_ (316 nm), which is in accordance with the difference in the chelating core of the two iron complexes, with the 1:3 species being a stronger complex. In all cases we should have an (*O*,*O*) coordination, occurring via *O*-phenol and *O*-methoxi, as already pointed out in the literature [48], thus explaining the lower values found for the copper and zinc complexation in comparison with iron, due to the *hard*/*soft* nature of Cu^2+^ and Zn^2+^ ions and the *hard* nature of Fe^3+^. The absence of full-coordinated metal–ligand species (ML_2_), in competition with the di-hydroxo-metal–ligand species (MH_−2_L) found for Cu and Zn systems, can be attributed to the low metal–ligand affinity and also to some hindrance of the methoxy groups to the metal–ligand interaction.

**Figure 3:**
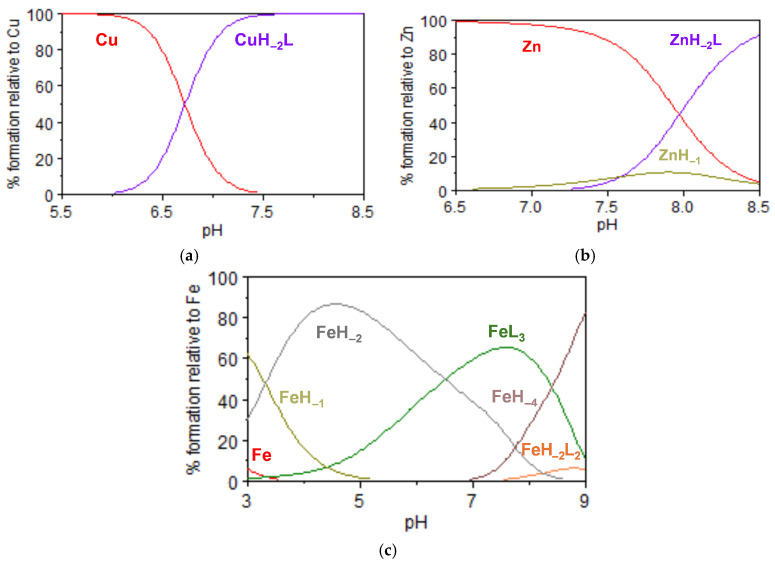
Species distribution curves for the systems (**a**) Cu^2+^/**4BY1** 1:2 (*C*_L_ = 4 × 10^−5^ M), (**b**) Zn^2+^/**4BY1** 1:2 (*C*_L_ = 6 × 10^−4^ M) and (**c**) Fe^3+^/**4BY1** 1:3 (*C*_L_ = 4 × 10^−5^ M).

The species distribution diagrams corresponding to the obtained metal complexation models are included in Figure 3 and were obtained under the experimental conditions used in the spectrophotometric titrations (Fe/L and Cu/L systems) and the potentiometric titrations (Zn/L system).

As expected from the experimental potentiometric data (see Figure 1), complex formation with **4BY1** begins at ca. pH 3 for iron, but only at ca. pH 6 and above 7 for copper and zinc, respectively (see Figure 3). Moreover, for all the metal/ligand systems, it is possible to observe competition with metal hydrolysis, with the formation of mixed metal–ligand–hydroxo species.

The pM values at the physiological pH (pH = 7.4; *C*_L_/*C*_M_ = 10; *C*_M_ = 10^−6^ M) can be used to compare the metal-chelating capacity of different ligands concerning diverse metal ions [49]. Therefore, the analysis in Table 1 allows us to establish a comparison with a different rivastigmine-derived hybrid already evaluated (RIV-IND), although care must be taken since different working media were used. Regarding RIV-IND, its metal chelation ability, especially for iron and copper, is higher than that of **4BY1**. Overall, the performed metal complexation studies, that to our best knowledge have never been covered in the literature for syringic acid or respective derivatives, allowed us to conclude that the rivastigmine hybrids derived from syringic acid studied herein have good and moderate–good chelating capacity for Fe^3+^ and Cu^2+^, respectively. So, the ability of this class of hybrids for copper chelation may aid the rationalization of the inhibitory capacity found for Aβ aggregation, namely as compared with non-chelating hybrids or stronger metal-chelating cinnamate derivatives. Furthermore, these hybrids should be able to modulate the iron and copper dyshomeostasis correlated with AD, therefore hitting one further pathophysiological target of this disease.

### 2.2. Inhibition of Aβ Self- and Copper-Induced Aggregation

Following our previous report on the in vitro biological properties of these new RIV hybrids, including their antioxidant and ChE inhibitory activity, as well as their capacity for Aβ_1–42_ aggregation [33], we have gone further on the assessment of other important biological properties, namely the inhibition of amyloid peptide aggregation. In particular, since, in our previous studies, it was shown that the compounds with higher inhibitory capacity for Aβ_1–42_ aggregation (**4AY5**, **4AY6**, and **4BY1**) contained metal-chelating groups, and since it is also known that elevated concentrations of biometal ions [15,50], in particular Cu^2+^, have been detected in Aβ plaques [51,52], we decided to complement the study of their capacity for inhibiting self-induced peptide aggregation with that in the presence of copper and potential Cu-induced Aβ_1–42_ aggregation. Therefore, the hybrids with metal-chelating moieties (**4AY1**, **4AY5**, **4AY6**, **4BY1**, and **4CY1**) were assayed by using the thioflavin T-based fluorometric method and curcumin as a positive reference.

Analysis of the results shown in Table 2 reveals that this series of RIV hybrids has moderate/good inhibitory potential towards Aβ self-aggregation (20–82%, for *C*_Inhibitor_ = 40 μM), with the highest values found for the hybrids bearing dihydroxycinnamate derivatives (**4AY5** and **4AY6**) or even syringic acid derivatives (**4BY1**), with the first two compounds showing a similar capacity to the reference curcumin (77%). The high inhibitory capacity of dihydroxycinnamate hybrids for Aβ self-aggregation is consistent with previously reported results for caffeic acid hybrids [35]. Therefore, these results point out the relevance of the chelating group, with two *ortho*-positioned hydroxyl substituents (**4AY5** and **4AY6**) or even a mono-hydroxyl group *ortho*-positioned with a methoxyphenolate (**4BY1**), in the self-inhibition of peptide aggregation, which is apparently associated with increased metal-chelating capacity, though other factors such as the large-sized moiety of the syringic acid derivatives (**4BY1**) may also exert some hinderance effect on the inhibitor–Aβ interaction strength and decrease its inhibitory capacity.

Analysis of the results obtained herein on the effect of the presence of Cu(II) on the capacity for Aβ-aggregation inhibition (see Table 2) shows good inhibitory capacity, namely for **4AY5** and **4AY6,** but, unexpectedly, does not show any increase in the inhibition for the syringic acid derivatives (**4AY1**, **4BY1** and **4CY1**) and thus apparently any relevance regarding inhibition of Cu-induced Aβ aggregation relative to the self-induced capacity previously reported [33]. The particularly low value found for **4CY1**, regarding the inhibition of Aβ aggregation in the presence of copper, can be explained by its low solubility in the experimental medium which led to the use of a lower concentration of inhibitor (20 μM). Therefore, although it is clear that the dihydroxycinnamate derivatives [35] and the hydroxy-methoxyphenolate studied herein, as well as the reference curcumin [53], are able to complex Cu(II), the role of these hybrids in the inhibitory capacity of Aβ aggregation remains unclear. Thus, the metal-chelating ability may have some role in the competition with Aβ for Cu(II), but other factors, such as the capacity of the compounds or their Cu(II) complexes for intercalation between β-sheets of Aβ fibrils, must also be considered in the inhibitory process. In fact, there is quite some controversy about the effect of metals, namely Cu(II), on the acceleration or retardation of Aβ fibrillation, which has been reported to be dependent on the higher or lower overall concentration of metal ions, as well as on Aβ aggregation kinetics [54], namely by formation of a ternary complex of copper, amyloid-β and the ligand [55]. So, the general slight decrease in the inhibition of Aβ aggregation in the presence of Cu(II) (see Table 2), relatively to Aβ self-aggregation, cannot be clearly rationalized but it may be due to kinetic retardation of the inhibition due to metal complex formation [56], though it is recognized that copper ion-specific chelators can reduce excess copper levels and mitigate copper-induced Aβ neurotoxicity in AD. Complementary studies by independent methods (e.g., AFM) will be further performed to aid the interpretation and rationalization of the results obtained herein.

### 2.3. Inhibition of hMAO-A and hMAO-B

The *h*MAO inhibitory activities of RIV-derived hybrids were evaluated by spectrophotometry, using kynuramine as a substrate and recombinant human MAO-A and MAO-B (*h*MAO-A and *h*MAO-B, respectively). Clorgyline and (*R*)-(–)-deprenyl were used as reference *h*MAO-A and *h*MAO-B inhibitors, respectively.

The results obtained showed that RIV-derived hybrids exhibit low inhibitory activities towards *h*MAO isoforms, with inhibition percentages not exceeding 50% at 10 µM (Table 2). In general, the highest percentages of *h*MAO inhibition at 10 µM were recorded for compounds containing cinnamic acid (**4AY3**, **4AY5**, and **4BY3**) or cinnamylidene-acetic moieties (**4AY4** and **4AY6**), ranging between 12.9 and 36.3% for *h*MAO-A and 6.6 and 43.1% for *h*MAO-B. The presence of a CH_2_ group between the rivastigmine-based ring and the cinnamoyl moiety resulted in increased inhibition of both *h*MAO isoforms (compound **4BY3** vs. **4AY3**). Moreover, catechols **4AY5** and **4AY6** displayed higher percentages of *h*MAO-A inhibition than the related methylenedioxyphenyl analogs **4AY3** and **4AY4**, respectively. On the other hand, the lowest *h*MAO inhibitory activities were found for compounds containing Trolox (**4AY2** and **4BY2**)- or syringic acid-based moieties (**4BY1** and **4CY1**).

Although in principle all the compounds should have the capacity to establish hydrogen bonds and hydrophobic and π-π interactions within *h*MAOs, the apparent major limitation may be their access to the enzymes’ active sites. To rationalize these results, docking modeling studies were carried out to shed some light on the different accessibilities of RIV-derived hybrids to the enzyme active site and their most important ligand–enzyme interaction patterns (see Section 2.4).

### 2.4. Molecular Docking Calculations Against hMAO-A and hMAO-B

Molecular docking was carried out to anticipate and rationalize the experimental inhibition data for both monoamine oxidase isoforms (Section 3.4) and to identify binding interaction patterns within the human enzyme active sites relevant to activity. Experimentally determined three-dimensional structures of human MAO-A and MAO-B were retrieved from the RCSB Protein Data Bank, screened according to predefined criteria (e.g., absence of mutations, high resolution, and the presence of a noncovalently bound active-site ligand), and subjected to computational evaluation. In the initial validation of the docking protocol, all selected structures were tested, and the performance of two docking programs, SMINA and GNINA, was assessed. Validation of the docking workflow by self-docking and cross-docking demonstrated that, for *h*MAO-A, PDB entry 2Z5X combined with SMINA provided the most consistent reproduction of the crystallographic pose, whereas for *h*MAO-B, 2XFN paired with SMINA was the best-performing configuration (Figure S1, Supplementary Materials). In the self-docking step, re-docking the crystallographic ligand into its native binding site, the top-ranked pose from 2Z5X/SMINA reproduced the experimental binding mode with an RMSD of 0.67 Å for *h*MAO-A. Likewise, for *h*MAO-B, the best pose obtained with 2XFN/SMINA yielded an RMSD of 0.49 Å. Additional self- and cross-docking results are provided in Figure S1 (Supplementary Materials). All RMSDs were computed with the *fconv* tool after aligning protein backbones to their crystallographic references.

Table 3 summarizes the predicted binding affinities (SMINA docking scores in kcal/mol) for each compound obtained with the validated docking protocol (more negative values indicate more favorable predicted binding). As expected, re-docking of the co-crystallized ligands (harmine for MAO-A and 2-(benzofuranyl)-2-imidazoline for MAO-B) accurately reproduces the crystallographic binding modes and produces strongly favorable predicted affinities (scores of −7.9 and −8.4, respectively), supporting the reliability of the docking workflow.

**Figure 4:**
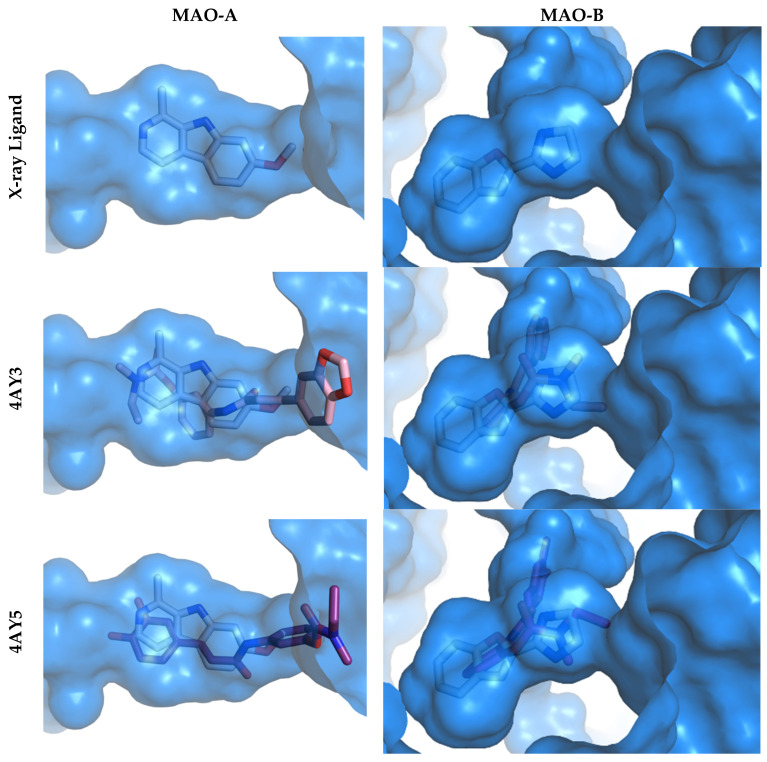

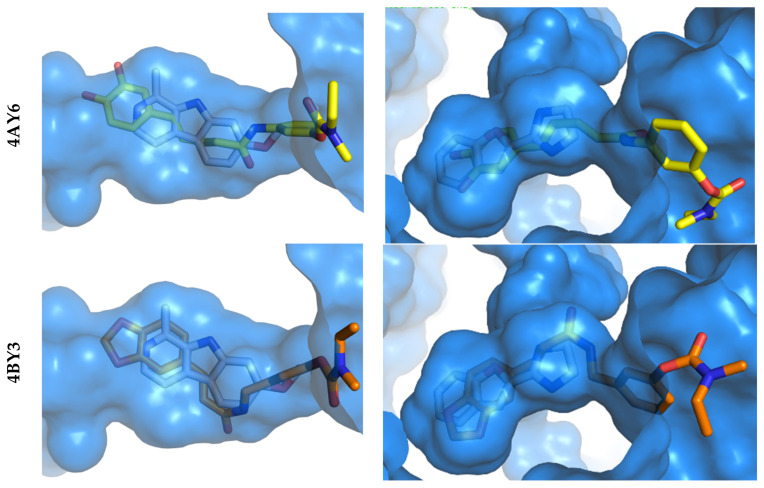
Representation of the best-binding poses of four of the studied compounds showing some of the highest inhibition values within the surface and binding pockets of MAO-A (**left**) and MAO-B (**right**). In all panels depicting the poses of the most active compounds, the corresponding crystallographic ligand is included for direct comparison. Compound **4AY3** is not among the four most active compounds against MAO-A, but it is included here because it is one of the most active against MAO-B and also shows inhibitory activity toward MAO-A.

**Figure 5:**
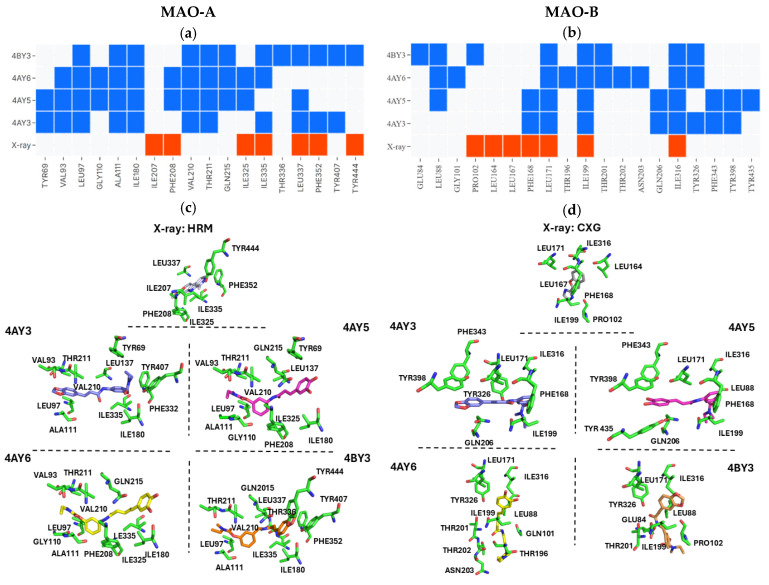
Enzyme–ligand interaction profiles and binding poses of the most active compounds compared with the co-crystallized ligand in monoamine oxidases. (**a**) MAO-A: interaction profiles for the most active compounds alongside the corresponding co-crystallized ligand; (**b**) MAO-B: interaction profiles, analogously; (**c**) representative binding poses of the most active compounds within the MAO-A binding pocket; (**d**) representative binding poses within the MAO-B binding pocket.

Across the compound set, docking scores are generally more favorable for MAO-B than for MAO-A. This trend is consistent with isoform-specific differences in active-site architecture. MAO-B contains a larger and bipartite binding cavity (entrance and substrate cavities) whose accessibility and effective shape are regulated by the Ile199/Tyr326 gating residues (Figure 4). PLIF analysis (Figure S3) indicates that all compounds (including **4AY3**, **4AY5**, **4AY6**, and **4BY3** in Figure 5) engage the MAO-B gate residue (Ile199) while also maintaining contacts along the entrance and channel regions, consistent with binding poses that span both cavities. More complete occupation of this extended binding space provides a structural basis for the more favorable MAO-B docking scores relative to MAO-A, which features a smaller, monopartite cavity and a distinct gating environment (e.g., Phe208/Ile335), thereby limiting the number and continuity of stabilizing contacts that can be formed.

For MAO-A, the docking scores range from 3.2 to −7.4 kcal/mol. Within the compound set, **4BY3** achieves a score of −7.4 kcal/mol, close to the score of harmine (−7.9) and consistent with its best-in-class experimental inhibition against MAO-A. Compounds **4AY1**, **4AY5**, **4BY1**, and **4CY1** exhibit intermediate scores between −6.0 and −6.5 kcal/mol.

For MAO-B, predicted affinities span from −3.0 to −9.0 kcal/mol (the least favorable value corresponding to **R4AY2**). The highest predicted affinities are observed for **4AY5** (−9.0 kcal/mol), **4AY3** (−8.9 kcal/mol), and **4BY3** (−8.1 kcal/mol), the latter also being the most potent experimental inhibitor in this set.

Although no unequivocal correlation was observed between docking scores and the percentage of inhibition for MAO-A and MAO-B, compound **4BY3**, which showed the highest inhibitory activity among the evaluated compounds, also presented the most favorable docking score for MAO-A (−7.4) and a score of −8.1 for MAO-B.

Analysis of the docking results further indicates stereochemical preferences within the MAO active sites. For compounds **4AY2** and **4BY2**, for which both R and S stereoisomers were evaluated, the S stereoisomer consistently displays more favorable docking scores against both enzyme isoforms. This trend aligns with the binding interaction patterns identified in the docking poses, in which the S configuration establishes more favorable contacts within the substrate cavity, providing a plausible structural rationale for the observed preference.

Figure 4 shows the top-ranked binding poses of the co-crystallized ligands together with representative active compounds **4AY3**, **4AY5**, **4AY6**, and **4BY3.** Compounds **4AY5**, **4AY6**, and **4BY3** show the highest inhibition values toward MAO-A, whereas compounds **4AY3**, **4AY5**, **4AY6**, and **4BY3** show the highest inhibition toward MAO-B. The complete set of top-ranked poses is provided in the Supplementary Materials (Figure S2).

Inspection of Figure 4 shows that compounds **4AY3**, **4AY5**, **4AY6**, and **4BY3**, which exhibit experimental activity toward both enzymes, consistently occupy the same binding pocket as the corresponding crystallographic ligands.

Notably, **4BY3**, which is the most active compound in the experimental assay, penetrates the deepest region of the binding pocket, closely reproducing the orientation and key interactions established by the crystallographic ligands in both isoforms. Owing to its larger size, **4BY3** also extends toward the pocket entrance, enabling substantial occupancy of this region, which is less extensively sampled by the co-crystallized inhibitor.

When examining the poses of the three most active ligands within the MAO-A binding pocket, we see that the compounds occupy the same general cavity. For compounds **4AY5**, **4AY6**, and **4BY3,** the cinnamic acid moiety is oriented toward the pocket entrance in contrast with **4AY3**. This difference may contribute to the lower inhibition value observed for **4AY3**. Compound **4AY5** aligns closely with the crystallographic ligand (harmine), yet due to its larger size, it can also extend toward the pocket entrance.

Among the compounds represented in Figure 4, **4AY5**, similarly to **4BY3**, reaches the deepest region of the pocket in MAO-A. In MAO-B, however, the presence of two hydroxyl groups (**4AY5** and **4AY6**) appears to limit its ability to form optimal interactions in the deepest region of the pocket, slightly displacing the ligand from the terminal portion of the cavity. Nevertheless, **4AY6** can still occupy the pocket entrance, resembling the behavior observed for **4BY3**.

Figure 5 presents the corresponding protein–ligand interaction profiles. This analysis reveals a clear conservation of key residues involved in ligand recognition across the docked compounds. For MAO-A, interactions with Phe208 and Ile325 are preserved between the crystallographic ligand and compounds such as **4AY5** and **4AY6**, while contacts with Ile335 are maintained for **4AY3**, **4AY6**, and **4BY3**. The presence of a catechol moiety in the leftmost portion of the pocket facilitates interactions with FAD, which is located there. Likewise, the benzodioxole moiety of compound **4BY3** is oriented toward FAD, enabling additional favorable contacts.

For MAO-B, the four most active inhibitors preserve the key interactions with residues Leu171, Ile199, and Ile316, mirroring the contacts observed for the co-crystallized ligand. In addition, compound **4BY3** establishes further interactions, engaging Glu84 and Leu88 along one side of the binding pocket and Thr201 and Tyr326 along the opposite side (Figure 5a–d). A complete set of enzyme–ligand interaction profiles for all docked compounds is provided in the Supplementary Material (Figure S3).

### 2.5. Cell Viability and Neuroprotection

The new RIV–antiox hybrids had already been tested for their protective role in the toxicity induced by either Aβ_1–42_ peptides or iron/ascorbate [33], and their effect on the toxicity induced by MPP^+^ was evaluated herein. SH-SY5Y cells were incubated with a set of concentrations of each compound, and a dose–response curve was performed to select the highest non-toxic concentration. This dose was found to be 1 µM for compounds **4AY2**, **4AY5**, **4BY2**, and **4BY3**; 2.5 µM for compound **4AY4**; 5 µM for compounds **4AY3**, **4AY6** and **4CY1**; and 10 µM for compounds **4AY1** and **4BY1**.

Despite the complexity of factors involved in AD pathogenesis, key hallmarks of AD etiology encompass the accumulation of senile plaques composed of Aβ aggregates, including the more toxic Aβ_1–42_ form found in the core of the plaque, along with ROS production [57]. Previously, we observed a significant decrease in cell viability following incubation with Aβ_1–42_ and iron/ascorbate of an average of 66% and 60%, respectively. Interestingly, compounds **4AY2** and **4BY2** were able to rescue both Aβ_1–42_-induced toxicity and toxicity induced by ROS after treatment with iron/ascorbate to levels that were not significantly different from untreated cells. Furthermore, compound **4AY4** showed a tendency to increase cell viability after the iron/ascorbate trigger.

Exposure to MPP^+^ causes neuronal loss through direct mitochondrial effects [58,59], which is linked with PD pathology, despite its complexity. Although MAO-catalyzed oxidative deamination generates hydrogen peroxide as a byproduct, and its inhibition may therefore limit one source of ROS formation, MAO activity alone cannot be considered a direct indicator of overall intracellular ROS modulation. For this reason, and because the present work was designed to characterize the compounds in terms of their multifunctional neuroprotective profile rather than to establish a specific intracellular antioxidant mechanism, direct ROS measurement was not included. In this context, MAO inhibition was evaluated as an independent and pharmacologically relevant property, given the well-established role of MAO, particularly MAO-B, in neurodegenerative disorders such as Parkinson’s disease and its potential relevance in Alzheimer’s disease. Assessing this activity is therefore important not only because of its effects on monoamine metabolism, but also because MAO inhibition may help reduce oxidative byproducts generated during MAO-catalyzed reactions.

**Figure 6:**
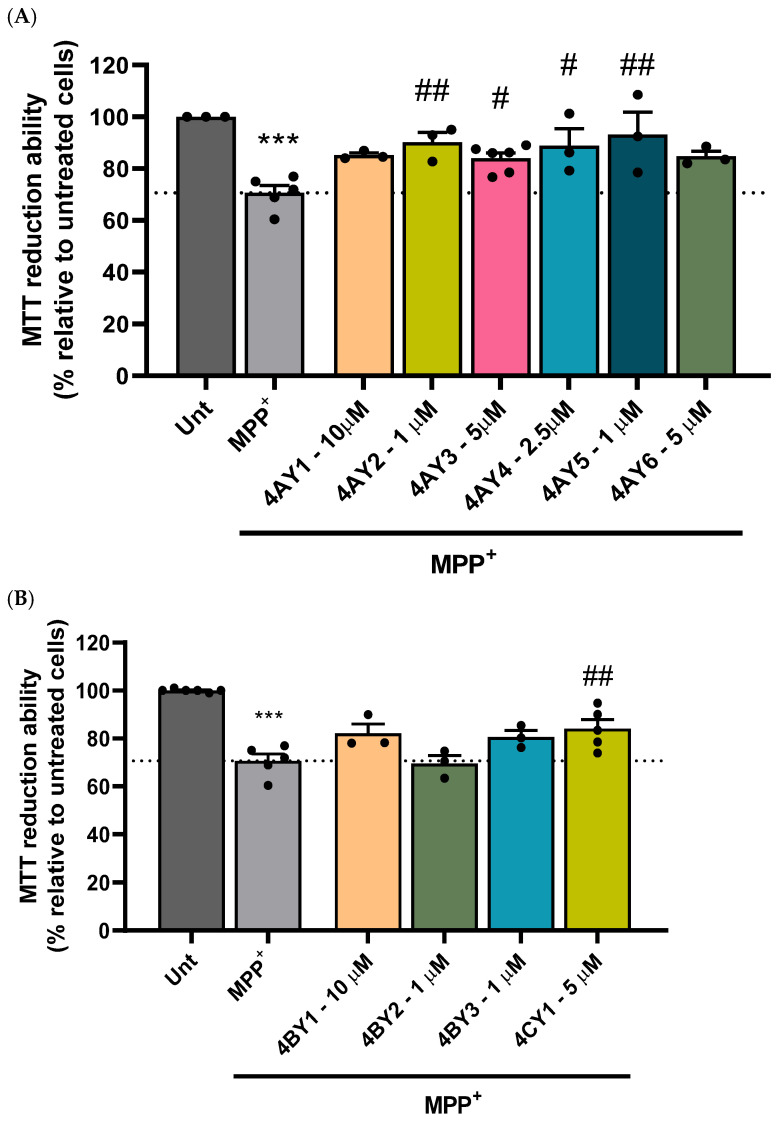
Neuroprotective effect of compounds (**A**) **4AY1–6** and (**B**) **4BY1–3** and **4CY1** against cellular toxicity induced by MPP^+^ measured by MTT reduction ability on SH-SY5Y cells pre-treated with the optimized concentration of each compound for one hour, followed by a 24 h incubation with MPP^+^. Results are expressed relative to untreated cells, with the mean ± standard error of the mean (SEM) derived from at least three independent experiments. *** *p* < 0.001 compared to untreated cells; # *p* < 0.05 and ## *p* < 0.01 compared to MPP^+^-treated cells. Statistical differences were analyzed using (**A**) one-way ANOVA followed by Dunnett’s multiple comparisons or (**B**) the Kruskal–Wallis test followed by uncorrected Dunn’s test in relation to untreated cells and unpaired Student’s *t*-test in relation to MPP^+^.

Consistent with previous findings, we observed a significant 30% decrease in cell viability after MPP^+^ incubation (Figure 6). Particularly, compounds **4AY4**, **4AY2**, **4AY5** and **4CY1** effectively rescued cells from MPP^+^-induced toxicity (Figure 6).

A limitation of the present study is that cell viability was assessed using a single assay reflecting cellular metabolic reduction capacity. Nevertheless, this parameter is relevant in the present model because MPP^+^ is known to target mitochondrial complex I and thereby disrupt mitochondrial function. Accordingly, the observed changes should be interpreted as indicative of altered metabolic viability rather than as a full mechanistic characterization of cell death. Further mechanistic studies are warranted to better define the pathways underlying these effects.

## 3. Experimental Section

### 3.1. Metal Chelation Assays

#### 3.1.1. Materials and Equipment

The hybrid compounds were synthesized according to the previously published procedure [33], involving a preliminary preparation of amino–RIV templates followed by their condensation with several commercially available functionalized aromatic carboxylic acids with antioxidant properties (antioxidant scaffolds).

The stock solutions of FeCl_3_ (0.0177 M), CuCl_2_ (0.015 M) and ZnCl_2_ (0.0156 M) were prepared from Titrisol standards (1000 ppm) and their metal content evaluated by flame atomic absorption spectroscopy. In order to prevent iron hydrolysis, the FeCl_3_ stock solution was prepared in acidic medium and the respective acid concentration was determined through the standard-addition method using a Titrisol 0.1 M HCl solution. The 0.1 M KOH titrant solution was standardized with potassium hydrogen phthalate and excluded whenever the percentage of carbonate exceeded 0.5% (Gran’s method [60]).

The glass electrode was formerly conditioned in 10% DMSO/water medium and the metal chelation studies were done in the same medium, by using an automatic potentiometric apparatus including a Crison micropH 2002 millivoltmeter, a Crison microBu 2031 burette and a Haake thermostatic bath (*T* = 25.0 ± 0.1 °C) controlled by the PASAT program. The spectrophotometric data was acquired with a Perkin-Elmer Lambda 35 spectrophotometer.

#### 3.1.2. Potentiometric and Spectrophotometric Titrations

The potentiometric and spectrophotometric titrations for the determination of the protonation and complex formation constants were accomplished in thermostatic glass cells (*T* = 25.0 ± 0.1 °C) and at an ionic strength (*I*) of 0.1 M KCl, by using 0.1 M KOH as a titrant. The potentiometric titrations of the ligand (**4BY1**) were performed in a total volume of 20 mL, the ligand concentration (*C*_L_) was 6 × 10^−4^ M and the metal-to-ligand molar ratios (*C*_M_/*C*_L_) were 0:1 (L), 1:1, 1:2 (M = Fe, Cu, and Zn) and 1:3 (M = Fe). The spectrophotometric titrations were accomplished in 30 mL, with *C*_L_ = 4 × 10^−5^ M, and the same *C*_M_/*C*_L_ ratios were used as for the potentiometric titrations involving the Fe/L and Cu/L systems. The spectra were carried out in a 250–370 nm wavelength range and for ca. pH 6–11 in the case of the ligand, ca. 3–11 for the Fe/L system and ca. 5–11 for the Cu/L system. All titrations were performed in duplicate.

#### 3.1.3. Calculation of the Constants

Under the chosen 10% DMSO/water medium, the calculated p*K*_w_ value was 13.8, which was further used in the computations. The stepwise protonation constants of the ligands, *K*_i_ = [H_i_L]/[H][H_i−1_L], and their overall metal–complex stability constants, $\beta_{M_{m}H_{h}L_{l}}$ = [M_m_H_h_L_l_]/[M]^m^[H]^h^[L]^l^ (M = Fe^3+^,Cu^2+^, and Zn^2+^), were calculated by fitting the spectrophotometric data with the Psequad program [61] and the potentiometric data with Hyperquad [62]. The following metal hydrolysis constants in water (*I* = 0.1 M KCl, *T* = 25.0 ± 0.1 °C) were used in the fitting analysis of the experimental data to the equilibrium models: log $\beta_{\mathrm{Fe}H_{-1}}$ = −1.99, log $\beta_{\mathrm{Fe}H_{-2}}$ = −5.3, log $\beta_{\mathrm{Fe}H_{-4}}$ = −20.8, log $\beta_{Fe_{2}H_{-2}}$ = −2.5, log $\beta_{Cu_{2}H_{-2}}$ = −10.98, log $\beta_{{Zn}_{H_{-1}}}$ = −8.6, log $\beta_{{Zn}_{H_{-3}}}$ = −27.2, and log $\beta_{{Zn}_{2}H_{-1}}$ = 8.6. The species distribution curves were obtained with the Hyss program [62].

### 3.2. Inhibition Assays of Aβ Aggregation

The ability of the RIV-derived hybrids to inhibit Aβ_42_ self- and Cu(II)-induced aggregation was evaluated by using a reported method based on the fluorescence emission of thioflavin T [63,64]. Stock solutions of 750 mg L^−1^ of the compounds were prepared in 50% MeOH/50% DMSO medium and afterwards, working solutions [240 μM (6% MeOH/6% DMSO) for all the ligands, but 120 μM (4% MeOH/4% DMSO) for **4AY4** and **4CY1**] of the hybrids were prepared by using appropriate volumes of phosphate buffer (0.215 M, pH = 8). A 0.149 mM Aβ_42_ (Shanghai Royobiotech Co., Ltd., Shanghai, China) solution was obtained by treatment with 1,1,1,3,3,3-hexafluoro-2-propanol (HFIP), after brief sonication and vortexing, and kept overnight at room temperature. After partition and evaporation, the Aβ_42_ aliquots were stored at −20 °C until needed. The Aβ_42_ working solution (40 μM) was prepared from the Aβ_42_ aliquots dissolved with a fresh mixture of 69.5 μL of CH_3_CN/Na_2_CO_3_ (300 μM)/NaOH (250 mM) (48.3/48.3/3.4, *v*/*v*/*v*), by brief sonication and vortexing, and then diluted with phosphate buffer. For copper-induced aggregation studies, a solution of CuCl_2_ 240 μM was prepared from a stock standard solution (0.015 M). Aβ_42_ (40 μM) was incubated at 37 °C for 24 h, with or without Cu(II) (40 μM), in phosphate buffer in the presence or absence of the single ligand [20 μM (0.7% MeOH/0.7% DMSO) or 40 μM (1% MeOH/1% DMSO)]. Afterwards, the samples with 180 μL of glycine–NaOH 50 mM buffer at pH = 8.50, containing 5 μM of ThT, were added to a 96-well microplate and fluorescence measurements were monitored in a fluorimeter with an excitation wavelength of 446 nm and an emission wavelength of 485 nm. Blank signals were subtracted from those of the corresponding samples. The fluorescence intensity of the Aβ_42_ + Cu(II) + RIV hybrid solutions was compared to that of the Aβ_42_ + Cu(II) solution to obtain the inhibition of the Cu(II)-induced Aβ_42_ aggregation for each hybrid. The reported values were obtained as the mean (SEM < 10%) of two different experiments in duplicate.

### 3.3. Inhibition of Monoamine Oxidases

The inhibitory activity of the RIV-based hybrids on *h*MAO-A and *h*MAO-B was studied using an experimental protocol described elsewhere [65,66]. The *h*MAO inhibition was assessed in microsomal MAO isoforms prepared from insect cells (BTI-TN-5B1-4) infected with recombinant baculovirus containing cDNA inserts for *h*MAO-A or *h*MAO-B (Sigma-Aldrich Quimica S.A., Toluca, Mexico) and by measuring the enzymatic conversion rates of kynuramine into 4-hydroxyquinoline. The appropriate amounts of *h*MAO-A and *h*MAO-B were adjusted to obtain, in our experimental conditions, the same maximum velocity (*V*_max_ = 50 pmol.min^−1^) for both isoforms (*h*MAO-A: 3 ng mL^−1^; *h*MAO-B: 12 ng mL^−1^). All assays were performed under sodium phosphate-buffered conditions (50 mM, pH = 7.4).

The compounds under study and reference inhibitors were pre-incubated at 37 °C for 10 min in the presence of kynuramine (*K*_m_ (*h*MAO-A) = 20 mM; *K*_m_ (*h*MAO-B) = 20 mM; final concentration: 2 × *K*_m_) in 96-well microplates (BRAND plates, pureGradeTM, BRAND GMBH, Wertheim, Germany). Then, the reaction was started with the addition of *h*MAO-A or *h*MAO-B. Initial velocities were determined spectrophotometrically in a microplate reader (Biotek Synergy HT) at 37 °C by measuring the formation of 4-hydroxyquinoline at 316 nm, over at least 30 min (intervals of 1 min). Data were analyzed using GraphPad PRISM version 8 for Windows (GraphPad Software^®^, San Diego, CA, USA). The initial velocities, obtained from the linear phase of product formation, were normalized to the control. For compounds with percentages of MAO inhibition > 50% at 10 µM, the percentages of MAO inhibition were plotted against the respective inhibitor concentrations. IC_50_ values were obtained from dose–response curves and were expressed as mean ± standard deviation. IC_50_ values were determined from at least three independent experiments, each performed in triplicate.

### 3.4. Molecular Modeling of MAO-A and MAO-B Inhibition

#### 3.4.1. Protein Structure Selection and Preparation of Protein and Ligand Models

Crystallographic structures of the target enzymes were selected based on three criteria: absence of point mutations relative to the canonical UniProt sequences, availability of high-resolution data, and co-crystallization with a small-molecule ligand in the active site. X-ray structures of human monoamine oxidase A (*h*MAO-A; UniProt ID: P21397; https://www.uniprot.org/uniprotkb/P21397/entry (accessed on 10 November 2025)) and human monoamine oxidase B (*h*MAO-B; UniProt ID: P27338; https://www.uniprot.org/uniprotkb/P27338/entry (accessed on 10 November 2025)) were retrieved from the RCSB Protein Data Bank (https://www.rcsb.org/ (accessed on 10 November 2025)). The initial filtering for molecular docking prioritized (i) the highest-resolution structures (lowest Å value) for each isoform to maximize coordinate accuracy and (ii) complexes in which a ligand was bound non-covalently in the substrate cavity adjacent to the covalently linked flavin adenine dinucleotide (FAD) cofactor, enabling reliable definition of the active-site conformation and docking grid. For *h*MAO-A, four structures were available, from which PDB entries 2Z5X and 2Z5Y were selected for subsequent calculations. For *h*MAO-B, PDB entries 2V5Z, 2XFN, and 6FWO met the selection criteria.

Protein and ligand preparation was performed in Molecular Operating Environment (MOE) version 2024.0601. Protein structures were imported from the PDB and processed by assigning bond orders, adding hydrogen atoms at pH 7.4, and correcting structural issues. The covalently bound flavin adenine dinucleotide (FAD) cofactor was retained in its native linkage to the active-site cysteine. To reduce computational complexity, crystallographic waters, buffer components, and non-essential chains and heteroatoms were removed unless identified as structural.

Co-crystallized ligands were extracted from the corresponding PDB entries. Synthesized compounds were constructed de novo in Maestro, and 3D structures were generated with the intended stereochemistry. All ligands were geometry-optimized, and their protonation and tautomeric states were assigned to reflect physiological conditions (pH 7.4) using MOE’s Protonate3D. Final ligand and protein energy minimizations were carried out in MOE with the Amber10:EHT force field to relieve local strain and obtain low-energy starting structures suitable for docking, using the software’s default convergence criteria.

#### 3.4.2. Molecular Docking Calculations and Analysis

Molecular docking was conducted with two independent software packages, SMINA (a fork of AutoDock Vina (version 1.1.2)) and GNINA (version 1.3, Vina with convolutional neural network-based scoring), to establish a robust protocol for pose prediction and ranking. For each protein structure, the binding site was defined by the coordinates of the co-crystallized ligand; the docking grid was centered on the ligand centroid and sized to encompass the substrate cavity and adjacent regions of the FAD cofactor. The covalently bound FAD was retained. Proteins were treated as rigid, and ligands were docked with full torsional and rotational flexibility. Unless otherwise specified, default search parameters were applied for both software packages, and multiple poses were generated per ligand.

Self- and cross-docking benchmarks informed the choice of the final protein structure and scoring function for prospective docking of the synthesized compounds. In self-docking, each co-crystallized ligand was removed from its native complex and re-docked into the corresponding binding site of the same protein to assess pose reproducibility. Pose accuracy was quantified as the ligand heavy-atom root-mean-square deviation (RMSD) between the top-ranked docked pose and the crystallographic reference, with RMSD ≤ 2.0 Å considered a successful reproduction of the experimental binding mode. In cross-docking, each ligand was docked into the other selected structures of the same isoform (*h*MAO-A or *h*MAO-B) to evaluate robustness across conformational variants. Performance was assessed by (i) RMSD relative to the cognate crystal pose and (ii) qualitative conservation of key active-site interactions, including orientation with respect to the FAD cofactor.

For *h*MAO-A, PDB entry 2Z5X (resolution 2.20 Å), combined with SMINA (affinity), yielded the most consistent pose reproducibility and ranking stability. For *h*MAO-B (at a resolution of 1.60 Å), 2XFN paired with SMINA was selected as the best-performing protocol. RMSDs were computed with the *fconv* tool after aligning protein backbones to their crystallographic references. In self-docking, the best RMSD values were 0.67 Å for *h*MAO-A and 0.49 Å for *h*MAO-B, meeting the commonly accepted ≤ 2.0 Å criterion for accurate pose reproduction (Figure S1, Supplementary Materials). As expected, cross-docking yielded higher RMSDs due to conformational differences among the crystal structures; nevertheless, key binding-site interactions and the orientation relative to FAD were qualitatively conserved, supporting the robustness of the selected protocol for prospective studies.

Protein–ligand interaction profiles were analyzed using the Protein–Ligand Interaction Fingerprints (PLIFs) Python library (PLIP) (version 2.3.0). Interactions assessed included hydrogen bonds (donor/acceptor-specific), hydrophobic contacts, π–π stacking, salt bridges, and halogen bonds.

### 3.5. Cell Viability and In Vitro Neuroprotection

Cell viability was assessed using the colorimetric MTT (3-(4,5-dimethylthiazol-2-yl)-2,5-diphenyltetrazolium bromide) assay, as described by Mosmann [67]. The compounds (**4AY1–6**, **3BY1–3**, and **4CY2**) were prepared in DMSO to create stock solutions at a concentration of 25 mM, which were then stored at −20 °C. A concentration range from 0.5 µM to 20 µM was screened to identify the highest non-toxic dose. The SH-SY5Y human neuroblastoma cell line (ATCC-CRL-2266) was grown in Dulbecco’s modified Eagle’s medium (DMEM) (Gibco-Invitrogen, Life Technologies Ltd., Paisley, UK) supplemented with 10% heat-inactivated fetal calf serum, 50 U/mL penicillin, and 50 µg/mL streptomycin at 37 °C in a 5% CO_2_ atmosphere. The cells were seeded at a density of 0.1 × 10^6^ cells/mL (when 48-well plates were used) or 0.15 × 10^6^ cells/mL (when 24-well plates were used) one day before the experiment commenced. Following the seeding, cells were treated with the selected concentrations of compounds for 24 h. After treatment, the cells were washed with PBS and incubated with 150 µL (in 48-well plates) or 200 µL (in 24-well plates) of MTT (0.5 mg/mL) for 2 h at 37 °C with 5% CO_2_. During this incubation, cellular dehydrogenases converted MTT into formazan crystals, which were subsequently dissolved in 150 µL (in 48-well plates) or 200 µL (in 24-well plates) of 0.04 M HCl/isopropanol, and absorbance was measured at 570 nm.

To evaluate the compounds’ protective effects against toxicity induced by MPP^+^, the cells were pre-treated with each compound for one hour before adding MPP^+^, followed by an additional 24 h incubation. MPP^+^ (1-methyl-4-phenylpyridinium, Sigma-Aldrich) was freshly prepared in sterile water on the day of the experiment and added at the final concentration of 0.5 mM. The concentrations tested for evaluating compounds’ potential protective role were: 1 µM for compounds **4AY2**, **4AY5**, **4BY2** and **4BY3**; 2.5 µM for compound **4AY4**; 5 µM for compounds **4AY3**, **4AY6** and **4CY1**; and 10 µM for compounds **4AY1** and **4BY1**. The final concentration of DMSO did not exceed 0.05% (*v*/*v*), and no significant changes in cell morphology were observed. Each plate included control groups with untreated cells as well as those exposed only to MPP^+^. The ability of compounds to reduce cell viability was normalized to that of untreated controls. Data are presented as mean ± SEM from at least three independent experiments conducted in duplicates. Statistical analyses were performed to assess normality using the Shapiro–Wilk test, followed by one-way analysis of variance (ANOVA) followed by Dunnett’s multiple comparisons, the Kruskal–Wallis test followed by uncorrected Dunn’s test (comparisons between untreated cells versus treatments), or unpaired Student’s t-test, with a significance threshold set at *p* < 0.05.

## 4. Conclusions

Due to multiple factors associated with the development and progression of neurodegenerative diseases (NDs), such as AD and PD, and the absence of an effective disease-modifying therapy, new strategies for the development of new small drugs based on a multi-target approach have been extensively implemented in recent years. A recent new set of multifunctional hybrids (RIV–antiox), resulting from fusing and linking the template of the rivastigmine drug with several antioxidant scaffolds (e.g., syringic acid, Trolox and cinnamic acid derivatives), demonstrated that several hybrids are able to conjugate the expected capacity for inhibition of both ChEs associated with the drug with antioxidant activity, inhibition of Aβ peptide aggregation, and also regeneration of neuronal cell death by AD-model insults. Based on these results, further studies have been pursued on the evaluation of other relevant biochemical properties of these hybrids. Specifically, assays were performed on the inhibition of copper-induced Aβ aggregation, on the capacity to modulate and interfere with other ROS-associated harmful dysfunctions, namely in the dysregulation of biometal ions (Fe^3+^, Cu^2+^, and Zn^2+^) or upregulation of monoamine oxidases (MAOs), and also on regeneration of neuronal cells from toxicity induced by a PD-model stressor (MMP^+^). These RIV hybrids revealed moderate/good inhibitory potential towards Aβ self- and Cu-induced aggregation, with the highest values found for the hybrids bearing dihydroxycinnamate derivatives (**4AY5** and **4AY6**) or syringic acid derivatives (**4BY1**), with the first two compounds showing similar inhibitory capacity to the reference curcumin. The quantitative evaluation of the metal-chelating capacity of **4BY1** with the redox-active metal ions Fe(III) and Cu(II) and also Zn(II) demonstrated that, similarly to the proposals for the other most active hybrids with cinnamate coordinating cores (**4AY5** and **4AY6**), this hybrid is capable of metal modulation, especially in iron and copper dyshomeostasis correlated with these NDs. In general, the highest percentages of *h*MAO inhibition were recorded for compounds containing cinnamate (**4AY3**, **4AY5**, and **4BY3**) or cinnamylidene moieties (**4AY4** and **4AY6**). In silico (docking simulations) studies aided the rationalization of structure–activity relationships related with the hybrids’ inhibitory capacity of these enzymes. Finally, the hybrids demonstrated a moderate/good capacity for rescuing neuronal-like cells from MPP^+^-induced toxicity, though higher effectiveness was showed for compounds **4AY4**, **4AY2**, **4AY5** and **4CY1**. Taking together the attained results, this set of hybrids with rivastigmine–antioxidant scaffolds seems to show a promising strategy for the development of multi-target drugs against NDs, such as AD and PD.

## Figures and Tables

**Table 1:** Protonation constants ^a^ of **4BY1** and RIV-IND for comparison as well as global formation constants ^b^ of their metal complexes (*T* = 25.0 ± 0.1 °C; *I* = 0.1 M KCl; 10% DMSO/water medium) and values of pM ^c^.

Compound	(mhl)	log *K*_i_	$\log\;\beta_{FeH_{h}L_{l}}$	$\log\;\beta_{{Cu}_{m}H_{h}L_{l}}$	$\log\;\beta_{{Zn}_{m}H_{h}L_{l}}$
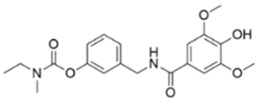 **4BY1**	(011)	8.627 (2) ^d^			
		8.61 (1) ^e^			
	(1-21)		-	−7.01 (7) ^d^	−11.88 (4) ^e^
				−6.31 (2) ^e^	
	(1-22)		5.27 (7) ^d^	-	-
			4.56 (8) ^e^		
	(103)		28.17 (8) ^d^	-	-
			25.56 (6) ^e^		
		**pM**	**15.8/15.6**	**7.5/8.2**	**6.0**
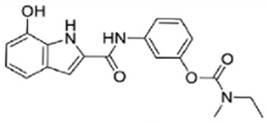 RIV-IND ^f^	(011)	9.40 (1)			
		9.85 (3)			
	(021)	3.30 (5)			
		2.38 (7)			
	(101)		12.23 (4)	-	-
	(1-11)		-	2.64 (5)	−1.70 (8)
	(102)		23.79 (6)	-	-
	(1-22)		-	−1.55 (6)	−4.2 (1)
	(103)		31.94 (8)	-	-
	(2-31)		-	−5.90 (8)	-
		**pM**	**17.0**	**9.0**	**6.3**

^a^ *K*_i_ = [H_i_L]/[H][H_i−1_L]; ^b^ $\beta_{MH_{h}L_{l}}$ = [M_m_H_h_L_l_]/[M]^m^[H]^h^[L]^l^; ^c^ pM = −log[M] at pH 7.4 (*C*_L_/*C*_M_ = 10, *C*_M_ = 10^−6^ M); ^d^ spectrophotometric measurements; ^e^ potentiometric measurements; ^f^ ref. [47], in 50% *w*/*w* DMSO/water.

**Table 2:** The inhibitory capacity of the RIV-derived hybrids (and reference compounds) for Aβ_42_ aggregation and monoamine oxidase (MAO).

Compound	Aβ_42_ Self-Aggr. Inhib. ^a,b^ (%)	Aβ_42_ Cu-Ind Aggr. Inhib. ^a^ (%)	% Inhibition at 10 µM	
			*h*MAO-A	*h*MAO-B
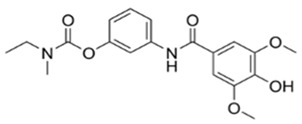 **4AY1**	20 ± 2	12.2	nd	nd
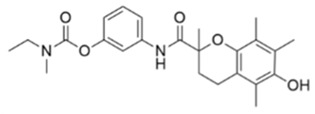 **4AY2**	45 ± 4	-	5.7%	4.1%
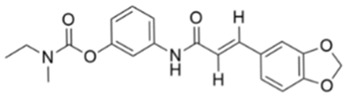 **4AY3**	45 ± 4	-	12.9%	25.5%
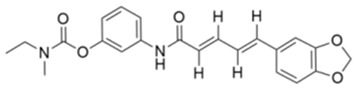 **4AY4**	23 ± 3	-	15.9%	6.6%
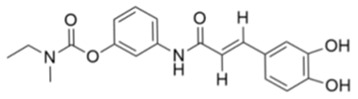 **4AY5**	82 ± 5	76.0	30.6%	16.9%
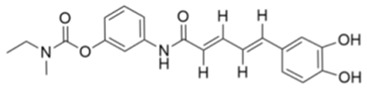 **4AY6**	75 ± 7	75.6	24.9%	16.9%
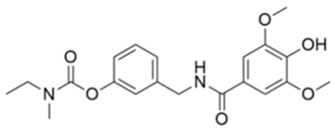 **4BY1**	58 ± 6	12.4	4.5%	4.1%
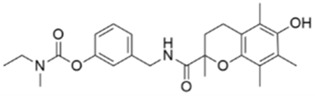 **4BY2**	47 ± 3	-	10.2%	8.3%
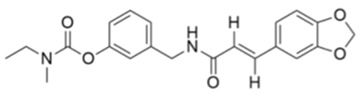 **4BY3**	29 ± 3	-	36.3%	43.1%
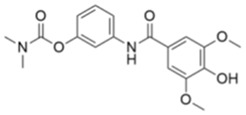 **4CY1**	31 ± 3	7.8	2.7%	6.6%
Curcumin	77 ± 1	53.0	-	
(R)-(–)-Deprenyl	-	-	16.3 ± 2.0	0.0494 ± 0.0060
Clorgyline	-	-	0.00257 ± 0.00036	2.94 ± 0.27

^a^ Percent of inhibition of Aβ_42_ aggregation in the presence or absence of copper (*C*_Cu_ = 40 μM; *C*_Inhibitor_ = 40 μM for all compounds but *C*_Inhibitor_ = 20 μM for **4AY4** and **4CY1**; thioflavin-T fluorescence method); the values are the mean of two independent measurements in duplicate (SEM < 10%). ^b^ Ref. [33].

**Table 3:** SMINA-predicted binding affinities (kcal/mol) for the RIV–antiox hybrids tested against human monoamine oxidase A (*h*MAO-A) and B (*h*MAO-B), and for the crystallographic ligands in the protein structures used (PDB ID: 2Z5X, harmine; PDB ID: 2XFN, 2-(2-benzofuranyl)-2-imidazoline).

Compound	SMINA Docking Scores	
	*h*MAO-A	*h*MAO-B
X-ray Ligand ^a^	−7.9	−8.4
**4AY1**	−6.0	−7.8
**R4AY2**/**S4AY2**	3.2/−5.0	−3.0/−5.3
**4AY3**	−4.1	−8.9
**4AY4**	−0.2	−7.7
**4AY5**	−6.1	−9.0
**4AY6**	−2.0	−8.0
**4BY1**	−6.6	−7.8
**R4BY2**/**S4BY2**	0.1/−4.1	−3.6/−7.7
**4BY3**	−7.4	−8.1
**4CY1**	−6.5	−7.3

^a^ harmine for MAO-A and 2-(2-benzofuranyl)-2-imidazoline for MAO-B.

## Data Availability

The original contributions presented in this study are included in the article/Supplementary Material. Further inquiries can be directed to the corresponding authors.

## Supplementary Materials

The following supporting information can be downloaded at: https://www.mdpi.com/article/10.3390/ijms27083637/s1.
