# Supplementary material for: Emerging Roles of Rivastigmine Derivatives Bearing Antioxidant Motifs as Multi-Target Agents for the Management of Neurodegenerative Diseases
Source: Int J Mol Sci. 2026 Apr 19;27(8):3637. doi: 10.3390/ijms27083637 (PMC13116261; doi:10.3390/ijms27083637)
Supplement: Supplementary file 1 [file ijms-27-03637-s001.zip › ijms-4232680-supplementary.pdf]

Supplementary Material

Emerging role of Rivastigmine derivatives bearing antioxidant motifs as multitarget agents for the management of neurodegenerative diseases

Inês Dias[1], Catarina Guerreiro-Oliveira [2,3,†], Inês Melo-Marques [2,3,†] Sandra M. Cardoso [2,3,4], Rita C. Guedes [5], Ismael Carvalho[5], Teresa Rocha[6], Daniel Chavarria[6], Sílvia Chaves [1,*] and M. Amélia Santos[1,*]

1 Centro de Química Estrutural, Institute of Molecular Sciences, Departamento de Engenharia Química, Instituto Superior Técnico, Universidade de Lisboa, Av. Rovisco Pais 1, 1049-001 Lisboa, Portugal

2 CNC-UC, Center for Neuroscience and Cell Biology, Universidade de Coimbra, 3004-504 Coimbra, Portugal; catarinaoliveira@cnc.uc.pt (C.G.-O.); ines.marques@cnc.uc.pt (I.M.-M.); sicardoso@fmed.uc.pt (S.M.C.)

3 Centre for Innovative Biomedicine and Biotechnology, University de Coimbra, 3004-504 Coimbra, Portugal

4 FMUC, Faculdade de Medicina, Universidade de Coimbra, 3004-504 Coimbra, Portugal

5 Research Institute for Medicines (iMed.ULisboa), Faculdade de Farmácia, Universidade de Lisboa, Av. Prof. Gama Pinto, 1649-003 Lisboa, Portugal; rguedes@ff.ulisboa.pt

6 RISE-Health, Department of Biomedicine, Pharmacology and Therapeutics Unit, Faculty of Medicine, University of Porto, Alameda Prof. Hernâni Monteiro, 4200-319 Porto, Portugal

a) b)

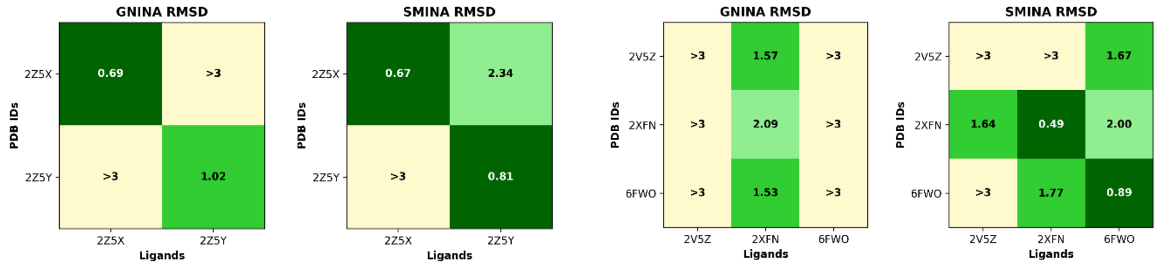

Figure SI.1. RMSDs calculated with the *fconv* tool for the best poses obtained from self-docking (diagonal) and cross-docking, performed to validate the docking protocol. Two docking programs (GNINA and SMINA) and five crystallographic structures were evaluated: a) two for MAO-A (2Z5X and 2Z5Y) and b) three for MAO-B (2V5Z, 2XFN, and 6FWO).

	**MAO-A**	**MAO-B**
**x-ray**	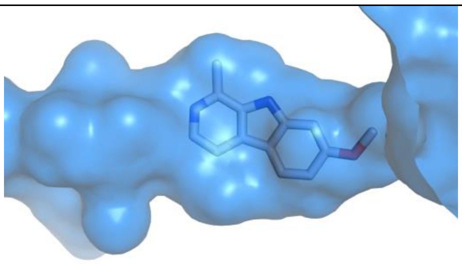	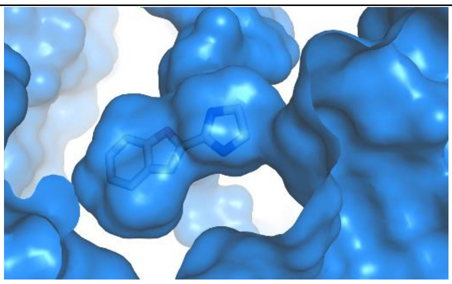

**4AY1**	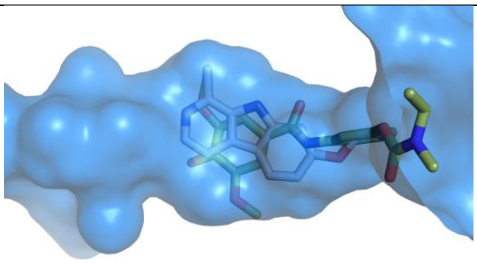	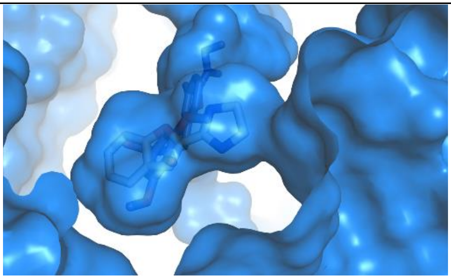
**4AY3**	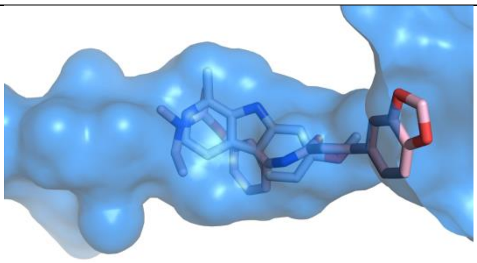	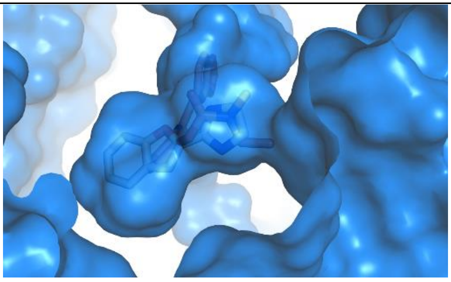
**R4AY2**	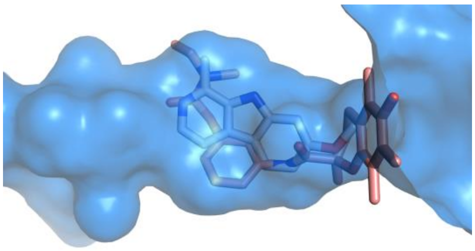	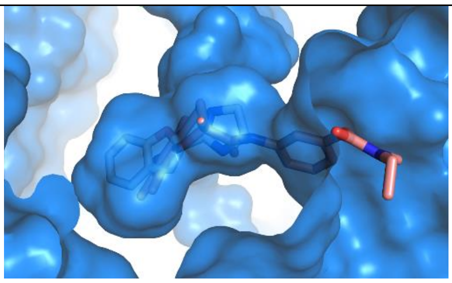
**S4AY2**	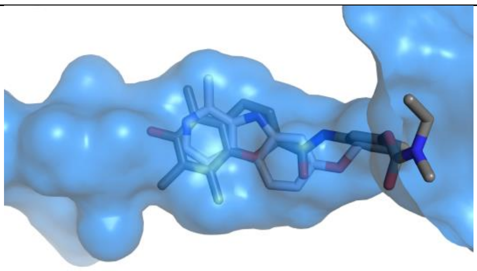	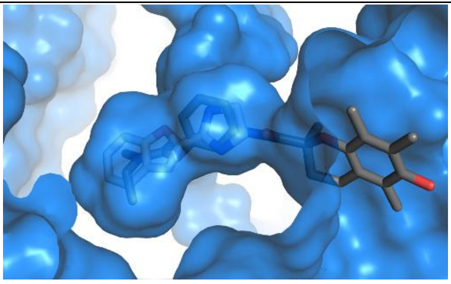
**4AY4**	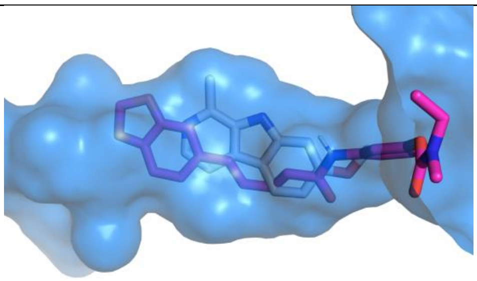	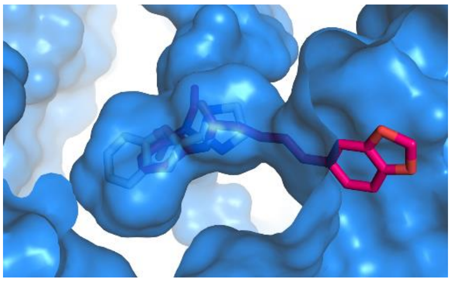
**4AY5**	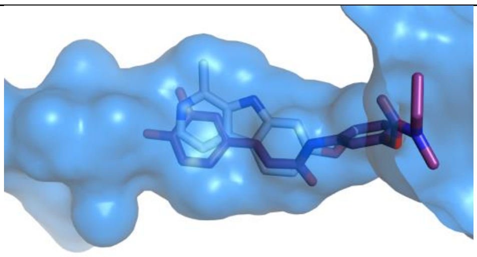	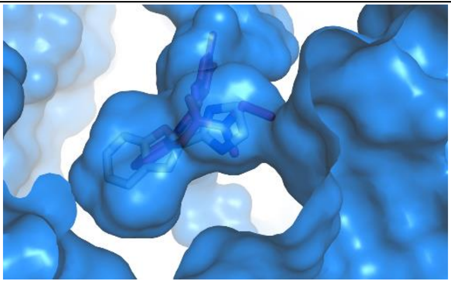

**4AY6**	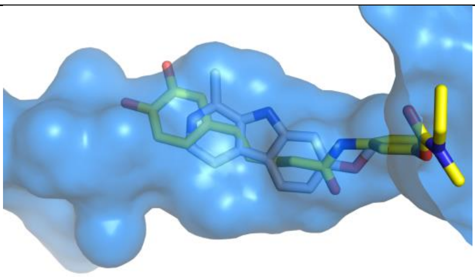	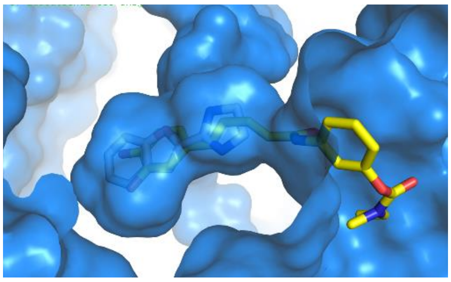
**4BY1**	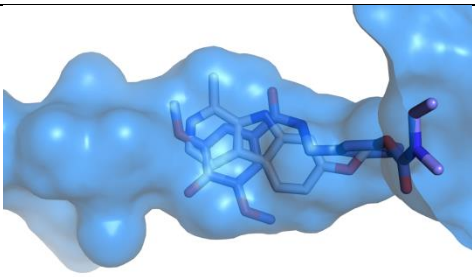	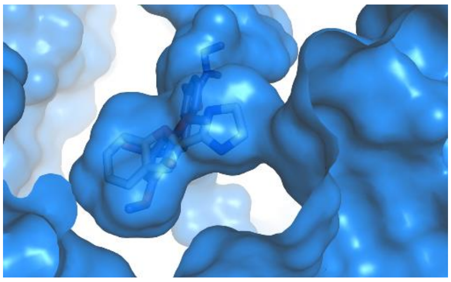
**R4BY2**	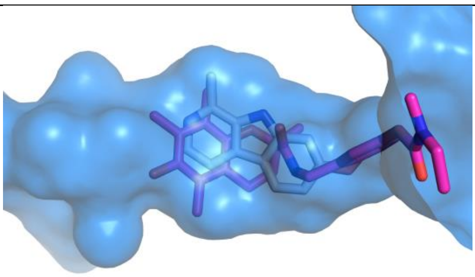	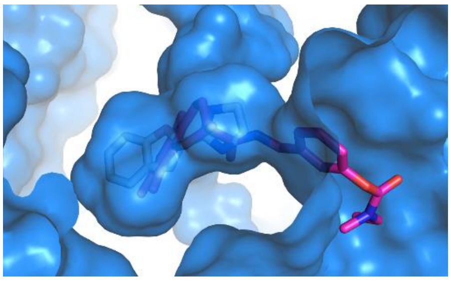
**S4BY2**	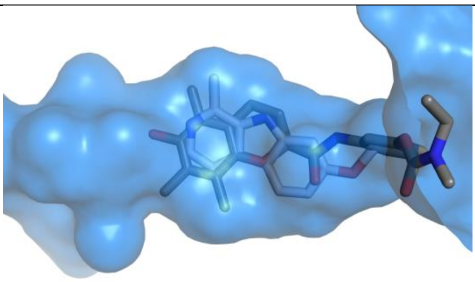	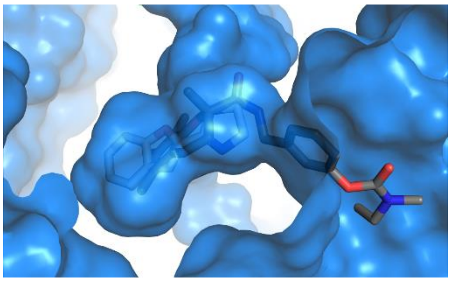
**4BY3**	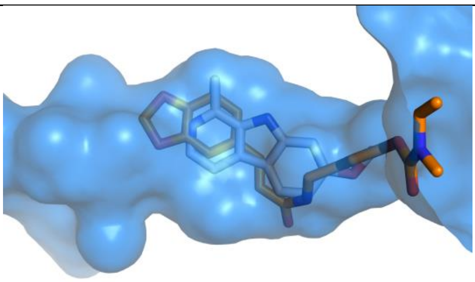	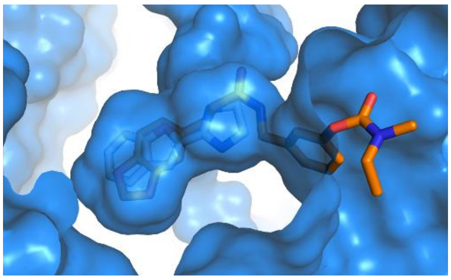
**4CY1**	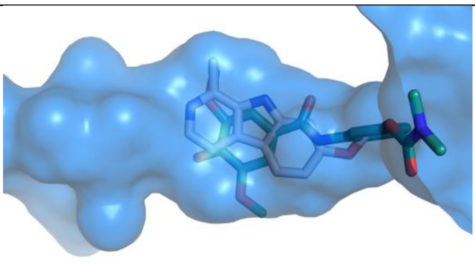	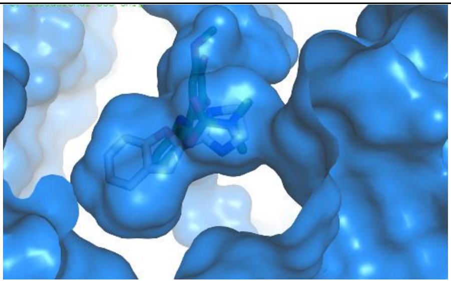

Figure SI.2. Representation of the binding poses of all studied compounds within the surface and binding pockets of MAO-A and MAO-B. In all panels depicting the poses of the most active compounds, the corresponding crystallographic ligand is included for direct comparison.

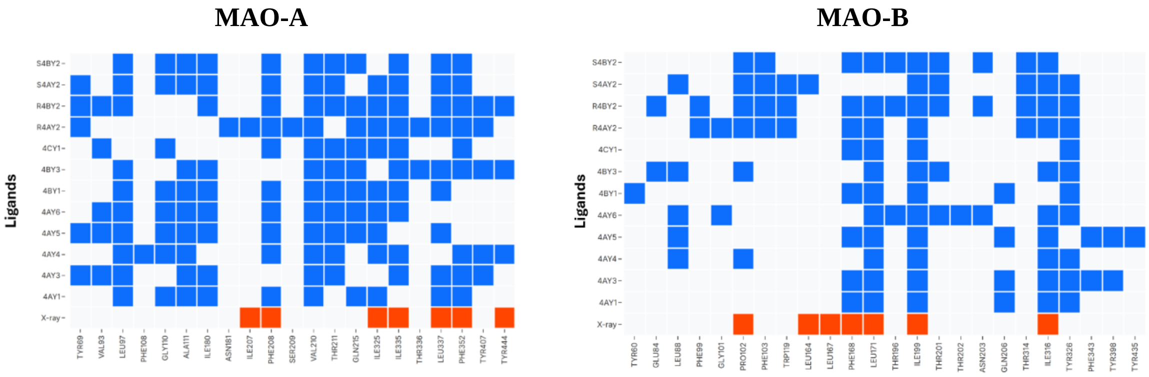

Figure SI.3. Enzyme–ligand interaction profiles and binding poses for all compounds studied compared with the co-crystallized ligand in monoamine oxidases. (a) MAO-A: interaction profiles for the most active compounds alongside the corresponding co-crystallized ligand; (b) MAO-B: interaction profiles
